# Incidence of cardiovascular events after kidney transplantation and cardiovascular risk scores: study protocol

**DOI:** 10.1186/1471-2261-11-2

**Published:** 2011-01-10

**Authors:** Salvador Pita-Fernández, Sonia Pértega-Díaz, Francisco Valdés-Cañedo, Rocío Seijo-Bestilleiro, Teresa Seoane-Pillado, Constantino Fernández-Rivera, Angel Alonso-Hernández, Dolores Lorenzo-Aguiar, Beatriz López-Calviño, Andres López-Muñiz

**Affiliations:** 1 Clinical Epidemiology and Biostatistics Unit, A Coruña Hospital, Hotel de Pacientes 7ª Planta, As Xubias 84, A Coruña, 15006, Spain; 2 Department of Nephrology, A Coruña Hospital, As Xubias 84, A Coruña, 15006, Spain

## Abstract

**Background:**

Cardiovascular disease (CVD) is the major cause of death after renal transplantation. Not only conventional CVD risk factors, but also transplant-specific risk factors can influence the development of CVD in kidney transplant recipients.

The main objective of this study will be to determine the incidence of post-transplant CVD after renal transplantation and related factors. A secondary objective will be to examine the ability of standard cardiovascular risk scores (Framingham, Regicor, SCORE, and DORICA) to predict post-transplantation cardiovascular events in renal transplant recipients, and to develop a new score for predicting the risk of CVD after kidney transplantation.

**Methods/Design:**

Observational prospective cohort study of all kidney transplant recipients in the A Coruña Hospital (Spain) in the period 1981-2008 (2059 transplants corresponding to 1794 patients).

The variables included will be: donor and recipient characteristics, chronic kidney disease-related risk factors, pre-transplant and post-transplant cardiovascular risk factors, routine biochemistry, and immunosuppressive, antihypertensive and lipid-lowering treatment. The events studied in the follow-up will be: patient and graft survival, acute rejection episodes and cardiovascular events (myocardial infarction, invasive coronary artery therapy, cerebral vascular events, new-onset angina, congestive heart failure, rhythm disturbances and peripheral vascular disease).

Four cardiovascular risk scores were calculated at the time of transplantation: the Framingham score, the European Systematic Coronary Risk Evaluation (SCORE) equation, and the REGICOR (Registre Gironí del COR (Gerona Heart Registry)), and DORICA (Dyslipidemia, Obesity, and Cardiovascular Risk) functions.

The cumulative incidence of cardiovascular events will be analyzed by competing risk survival methods. The clinical relevance of different variables will be calculated using the ARR (Absolute Risk Reduction), RRR (Relative Risk Reduction) and NNT (Number Needed to Treat).

The ability of different cardiovascular risk scores to predict cardiovascular events will be analyzed by using the c index and the area under ROC curves. Based on the competing risks analysis, a nomogram to predict the probability of cardiovascular events after kidney transplantation will be developed.

**Discussion:**

This study will make it possible to determine the post-transplant incidence of cardiovascular events in a large cohort of renal transplant recipients in Spain, to confirm the relationship between traditional and transplant-specific cardiovascular risk factors and CVD, and to develop a score to predict the risk of CVD in these patients.

## Background

Cardiovascular disease (CVD) is one of the most common complications after renal transplantation, with a considerably higher incidence than in the general population [1]. Although in recent years some authors have documented a significant reduction in cardiovascular death after kidney transplantation [2], today CVD is still the major known cause of death in kidney transplant patients [3].

In addition to the conventional CVD risk factors (such as obesity, smoking habits, diabetes, hypertension or dyslipidemia), several other factors seem to influence the high incidence of cardiovascular events in renal transplant patients [4,5]. These include, amongst others, the duration of prior dialysis [6-8], graft function after transplantation [9-11], hyperhomocysteinemia [12-16], elevated inflammatory markers [16-18], proteinuria [19-22], acute rejection episodes [23], post-transplant diabetes mellitus [6,24-27], and the toxic effects derived from the use of immunosuppressant medications and other drugs [28,29].

Therefore, when evaluating patients prior to kidney transplantation, special attention should be given to cardiovascular risk factors. To determine the individual risk of cardiovascular events, risk scores have been established in general populations, such as the Framingham Heart Study score [30,31], the Regicor (Framingham calibrated to Spain) [32], SCORE [33] or DORICA [34]. However, the validity of these equations in more selected populations, such as those of transplant patients, is not well established. In renal transplant recipients, the Framingham score does not seem to accurately predict the individual cardiovascular risk, leading to an underestimation of the cardiovascular event rates [4,16]. A recent study has corroborated the poor predictive accuracy of the Framingham score in these patients, indicating the need to consider new predictive models that incorporate both traditional and non-traditional cardiovascular risk factors [35].

Determining the incidence of cardiovascular events after a kidney transplant and the associated risk factors is therefore important in order to inform physicians of the need for CVD screening and prevention as part of the transplant evaluation [36]. Moreover, an accurate estimation of a patient's risk of cardiovascular disease could make it possible to identify people at high risk of suffering a cardiovascular event, and intervene proactively before they develop the disease.

This project was designed with three main objectives: firstly, to determine the incidence of post-transplant cardiovascular events after renal transplantation and related factors; secondly, to examine the ability of established cardiovascular risk scores (Framingham, Regicor, SCORE, and DORICA) to predict post-transplantation cardiovascular events in renal transplant recipients; and finally, to develop and validate a cardiovascular risk score to predict the risk of developing a cardiovascular event after kidney transplantation.

## Methods/Design

### Design and study population

Observational, retrospective and prospective cohort study of all adult (> 18 years) patients who underwent primary or repeated kidney transplantation with a graft from a cadaveric or living donor in the A Coruña Hospital (northwest Spain) between 1 January 1981 and 31 December 2008. Patients who had undergone simultaneous kidney-pancreas transplantation will be excluded from the analysis.

### Measurements

All the variables that will be recorded for each one of the patients included in the study are summarized in Additional file 1: Table S1.

Characteristics recorded at the time of transplantation include donor and recipient age and gender, body weight, height, body mass index (weight in kilograms divided by the height in meters squared), primary cause of renal failure (type I diabetes mellitus, type II diabetes mellitus, primary glomerulonephritis, nephrosclerosis including hypertension and renovascular disease, polycystic kidney disease, systemic lupus erythematosus, and others), cold ischemia time, type of dialysis prior to transplant (hemodialysis or peritoneal dialysis), total duration of renal replacement therapy before transplantation, and previous transplants. The date of transplantation and the type of donor (a deceased or living donor) will also be recorded.

Information about other traditional cardiovascular risk factors at the time of transplantation will also be collected, including history of tobacco smoking, hypertension, hypercholesterolemia, left ventricular hypertrophy, prior diabetes and a history of cerebrovascular disease (transient ischemic attacks and strokes), peripheral vascular disease (revascularization procedures and amputations) and malignancies prior to transplantation.

After transplantation, episodes of acute rejection and infections will be registered, together with the patient's smoking status, new-onset diabetes and diagnosis of left ventricle hypertrophy. Biochemical parameters will be collected during the post-transplantation follow-up at week 1, week 2, month 1, month 3, month 6, and yearly thereafter. These will include white blood counts, hematocrit, haemoglobin, cholesterol, high-density lipoprotein (HDL) cholesterol, low-density lipoprotein (LDL) cholesterol, triglycerides, creatinine levels and 24-hour proteinuria. At the same time intervals, systolic and diastolic blood pressure will be recorded. Renal function will be estimated using the Cockcroft-Gault formula [37] and the modified Modification of Diet in Renal Disease (MDRD) equation [38]. In the analysis of factors predicting new post-transplant cardiovascular events, the average of laboratory and blood pressure values recorded before the event will be considered.

For the analysis, the cut-points defining the control of each risk factor will be taken from the European Guidelines on CVD prevention [39]. For estimates of dyslipidemia control based on LDL, HDL cholesterol and triglycerides, we will use the cut-points agreed in the consensus statement from the American Diabetes Association and the American College of Cardiology Foundation [40].

The initial immunosuppressive regimen of the patients and changes in treatment during follow-up will be recorded. Dosage of immunosuppressive drugs will also be registered after transplantation at week 1, week 2, months 1, 3 and 6, and yearly thereafter. The use of antihypertensive and lipid-lowering drugs after transplantation will also be registered at the same times.

Post-transplant cardiovascular events are defined as the presence of myocardial infarction, invasive coronary artery therapy, cerebral vascular events (stroke and transient ischemic attacks), new-onset angina, congestive heart failure, rhythm disturbances (ventricular tachycardia, atrial fibrillation and the need for a pacemaker), and peripheral vascular disease. Peripheral vascular disease is defined as the need for invasive therapy or conservatively managed limb gangrene.

Patients will be followed-up from the date of renal transplantation until death or their return to dialysis.

### Quantification of cardiovascular risk

Four established cardiovascular risk scores will be applied to the kidney transplant recipients at the time of transplantation: the Framingham risk factor score, the European Systematic Coronary Risk Evaluation (SCORE) equation, and two coronary risk equations that use the calibrated Framingham functions for the Spanish population, the REGICOR (Registre Gironí del COR (Gerona Heart Registry)) and DORICA (Dyslipidemia, Obesity, and Cardiovascular Risk) functions.

The Framingham risk score will be calculated using both the classic Framingham-Anderson equation [30] and the equation modified by Wilson [31]. These scores predict the risk of developing fatal or non-fatal coronary heart disease (CHD) within a 10-year time period in adults between the ages of 30-74 with no known previous history of CHD, stroke, or peripheral vascular disease. CHD includes angina pectoris, myocardial infarction, coronary insufficiency (unstable angina) and coronary heart death.

The original Framingham-Anderson equation includes the following risk factors: age, sex, systolic blood pressure, total cholesterol, HDL-cholesterol, diabetic status, tobacco smoking status and evidence of left ventricular hypertrophy on resting electrocardiogram (ECG). The Framingham-Wilson equation replaces the continuous measurements of systolic blood pressure, total cholesterol and HDL-cholesterol with categorical variables based on the risk categories of the Joint National Committee on Blood Pressure and the National Cholesterol Education Program (NCEP) [41-43]. It also takes into account information regarding diastolic and LDL-cholesterol levels, and does not include the diagnosis of left ventricular hypertrophy due to its high association with hypertension and the absence of standard, universally accepted ECG criteria.

The European SCORE was developed by the European Society of Cardiology in 2003 as an estimation of 10-year risk of fatal cardiovascular disease (CVD) in subjects between the ages of 24 and 80. The variables used to calculate this score include age, gender, total cholesterol, systolic blood pressure and current smoking status [33]. The main difference between the SCORE risk chart and the Framingham model is that it estimates the risk for all atherothrombotic cardiovascular manifestations, including stroke, heart failure, peripheral arterial insufficiency or certain aneurysms, and not just CHD. The SCORE charts are currently the CVD charts recommended by the European Societies [44] and the Spanish Interdisciplinary Committee for Cardiovascular Disease Prevention (CEIPC) [45]. The SCORE risk charts calibrated for Spain will be used [46].

Finally, the REGICOR [32] and the DORICA [34] charts are adaptations of the Framingham equation to the Spanish population characteristics. Like the Framingham equation, these functions estimate the risk of coronary mortality and morbidity using information about age, gender, total cholesterol, HDL-cholesterol, systolic and diastolic blood pressure and smoking habits, differentiating diabetic from non-diabetic patients.

Patients will be classified as high-risk patients when their ten-year risk of coronary heart disease is ≥20% according to Framingham [31], REGICOR [32] and DORICA [34] equations or when their ten-year risk of cardiovascular mortality is ≥5% according to the SCORE system [44].

### Sample size

During the study period (1981-2008) n = 2059 kidney transplants were performed in the A Coruña Hospital, corresponding to 1794 patients.

This sample size will make it possible to detect as significant, in a Cox regression model, a relative risk of 1.33 or more associated with a prevalence of exposure to a cardiovascular risk factor of 50% and a censored data percentage of 78% (Security: 95%; Statistical power: 80%). Assuming an exposure prevalence of 50% maximizes the sample size necessary to detect a relative risk of this magnitude. According to previously published data for this cohort of patients, the estimated incidence of CVD at five years is 22.2% [47], and so the censoring value was estimated at 78%. In this situation, the sample size required to estimate a relative risk of 1.33 or more (α = 0.05, β = 0.2) would be n = 1755 patients.

### Statistical analysis

A descriptive analysis of the variables recorded will be performed. Observed frequencies and percentages will be used for qualitative variables, with their 95% confidence interval, and the mean and standard deviation or median and interquartilic range for quantitative variables.

The primary outcome for this study will be the estimation of the probability of cardiovascular disease in the presence of other competitive events in renal transplant patients. The cumulative incidence of cardiovascular events after kidney transplantation will be analyzed by competing risk survival methods, where each subject is at risk of failure from different causes. Therefore, each patient will be classified in four states of interest (alive with functioning graft, death, graft failure and cardiovascular disease).

Cumulative incidence functions will be used to compare the observed incidence of events between groups [48]. The cumulative incidence function regression model of Fine and Gray will be used for multiple regression analyses [49]. Additionally, the clinical relevance of different variables will be evaluated using the ARR (Absolute Risk Reduction), RRR (Relative Risk Reduction) and NNT (Number Needed to Treat).

In order to analyze the ability of each of the four cardiovascular risk scores to predict cardiovascular events rates, the concordance c index [50] and the area under the receiver-operating characteristic (ROC) curves, using a time-dependent approach [51], will be calculated. To calibrate the scores, relative risk ratios for observed-to-predicted event rates will be computed, and the Hosmer-Lemeshow test will be applied.

Based on the competing risks analysis, a nomogram to estimate the probability of cardiovascular events after kidney transplantation will be developed. The objective is to use the conditional cumulative incidence function to provide a patient-specific prediction of the probability of failure in the setting of competing risks.

The predictive performance of the nomogram will be evaluated by assessing both discrimination and calibration values [52]. A bootstrap re-sampling approach with 1000 replications will be used to reduce overfit bias. Discrimination will be quantified with the concordance c index and the area under the receiving operating characteristic (ROC) curve [50,51]. The nomogram will then be calibrated graphically, comparing the predicted probabilities with the actual rate of patients with cardiovascular events. A Loess curve will be fitted to the data and compared with an ideal fit. In addition, the Brier score will be calculated as a measure of the predictive accuracy of the model.

A p-value of < 0.05 will be considered as statistically significant (two-sided). Statistical analyses will be carried out with SPSS version 17.0 (SPSS Inc., Chicago, IL.) and R version 2.9.1 (The R Foundation for Statistical Computing), adding the ROCR, Design and Hmisc libraries.

### Ethics

The study has received written approval from the regional Ethics Committee for Clinical Research (CEIC Galicia) (code 2007/019). Informed consent will be obtained from all the participants in the study.

## Discussion

In recent years, several important changes have occurred in the management of kidney transplant patients that have been associated with an improvement in rejection rates and early graft survival [53]. Therefore, attention is now focusing on improving long-term transplant outcomes [3]. Renal dysfunction and increased cardiovascular risk are some of the factors that have a negative impact on long-term graft and patient survival. Although some authors have documented a reduction in CVD-related deaths in kidney transplant recipients in recent years, CVD is still the leading cause of death in patients with a functioning allograft [2].

Several studies have analyzed the risk factors connected with the incidence of cardiovascular events after kidney transplantation [4-29]. However, the role of some novel risk factors remains unclear. Furthermore, most of the previous studies are based on retrospective analyses of registry data, which contains information only on baseline data at the time of transplantation and the immediate post-transplantation follow-up. As a result, long-term follow-up data on clinical, biochemical and treatment covariates after transplantation is not always available.

This study will allow us to determine the post-transplant incidence of cardiovascular events in a cohort of renal transplant recipients in Spain, as well as to confirm the relationship between some CVD risk factors and the occurrence of a cardiovascular event in these patients. The development of a specific CVD risk score for kidney transplant recipients would make it possible to identify patients at high risk for coronary events at the time of transplantation, or shortly after the transplant. Therefore, this risk assessment could be used to recommend lifestyle changes for patients, to initiate appropriate treatment, or to redesign the immunosuppressive regimens for the prevention of primary or recurrent cardiovascular events in kidney transplant patients. The majority of studies use survival analysis strategies with the methodology of Kaplan and Meier. As this methodology may lead to overestimating the likelihood of the occurrence of the events of interest, we propose a competitive risk analysis in order to study the prognosis of these patients [48,49].

One of the limitations of this study is that information will mostly be taken retrospectively from hospital records, which could lead to information bias. On the other hand, transplant recipients are subjected to a more exhaustive follow-up than normal, not only during the immediate post-transplant period, but also during the years after the transplant. This fact would minimize any possible information bias. Likewise, during the study period standard immunosuppressive therapy has changed, and the treatment for each patient will depend on the date of transplantation and other medical subjective criteria. In order to control the changes that have occurred over the years, adjustments will be made based on the year of transplantation. Also, the therapeutic changes that occurred during the follow-up period for each of the patients will make it difficult to establish relationships between the medication and the cardiovascular events. Therefore, the impact of immunosuppressant medication on the presence of cardiovascular events in the follow-up could not be well established. The role of immunosuppressant agents must be investigated in greater detail with randomized clinical studies, and not with observational studies of this kind. However, our study will include a large cohort of patients with a long follow-up period, and the sample size is expected to be large enough both to identify individual CVD risk factors from multivariate analysis and to develop a new CVD prediction model.

## Competing interests

The authors declare that they have no competing interests.

## Authors' contributions

SPF, SPD, FVC and RSB participated in the design and coordination of the study. SPD, TSP and BLC are the biostatisticians of the study. SPF, SPD, FVC, RSB, CFR, AAF, DLA, BLC and ALM reviewed the study protocol and made suggestions that improved the design. All of these individuals are involved in the management of the study. SPF and SPD drafted the manuscript. All of the authors read, revised and approved the final manuscript.

## Pre-publication history

The pre-publication history for this paper can be accessed here:

http://www.biomedcentral.com/1471-2261/11/2/prepub

## Supplementary Material

Additional file 1 **Table S1, Study measurements**. Table displaying the measurements that will be recorded on each patient at the time of transplantation and during the follow-up. Click here for file
