# Supplementary material for: Incidence of cardiovascular events after kidney transplantation and cardiovascular risk scores: study protocol
Source: BMC Cardiovasc Disord. 2011 Jan 10;11:2. doi: 10.1186/1471-2261-11-2 (PMC3022886; doi:10.1186/1471-2261-11-2)
Supplement: Additional file 1 — Table S1, Study measurements. Table displaying the measurements that will be recorded on each patient at the time of transplantation and during the follow-up. [file 1471-2261-11-2-S1.DOC]

**Supplementary Table 1. Baseline and post-transplant study measurements.**

| - Year of transplantation |
| --- |
| **Donor variables** |
| - Age (years) - Gender - Type of donor (deceased versus living) |
| **Recipient variables** |
| **Sociodemographic variables** |
| - Age (years) - Gender |
| **Chronic kidney disease-related risk factors** |
| - Primary renal disease - Renal replacement therapy before transplantation - Duration of renal replacement therapy before transplantation - Previous transplant - Cold ischemia time |
| **Pre-transplant cardiovascular risk factors** |
| - Weight (kg), height (m) and body mass index (kg/m^2^) - Pre-transplant systolic and diastolic blood pressure (BP) - Pre-transplant cholesterol (mg/dl), high-density lipoprotein (HDL) cholesterol (mg/dl), low-density lipoprotein (LDL) cholesterol (mg/dl) and triglycerides (mg/dl) - Cardiovascular events before transplantation - Previous malignancies - Smoking status at transplantation (current smoker, former smoker or never smoked) - Diabetes mellitus pre-transplantation - Left-ventricular hypertrophy |
| **Post-transplant cardiovascular risk factors** |
| - Body mass index (kg/ m^2^) - Post-transplant systolic and diastolic blood pressure (BP) - New-onset diabetes mellitus after transplantation - Smoking status post-transplantation - Post-transplant left-ventricular hypertrophy |
| **Routine biochemistry on follow-up** |
| - Creatinine (mg/dl) - Proteinuria (g/day) - Leukocytes (number/L) - Hematocrit (%) - Haemoglobin (g/dl) - Serum albumin (g/dl) - Total cholesterol (mg/dl), HDL-cholesterol (mg/dl), LDL-cholesterol (mg/dl) and triglycerides (mg/dl) |
| **Treatment** |
| - Immunosuppressive therapy - Antihypertensive agents - Lipid-lowering drugs |
| **Endpoints in the follow-up** |
| - Post-transplant cardiovascular events - Myocardial infarction - Invasive coronary artery therapy - Cerebral vascular events - New-onset angina - Congestive heart failure - Rhythm disturbances (ventricular tachycardia, atrial fibrillation, need for a pacemaker) - Peripheral vascular disease - Acute rejection episodes - Graft failure - Death |
